# Correlation of Glucose Metabolism with Cancer and Intervention with Traditional Chinese Medicine

**DOI:** 10.1155/2022/2192654

**Published:** 2022-10-14

**Authors:** Gui-hua Lai, Fei Wang, Duo-rui Nie, Shu-jun Lei, Zhuo-jun Wu, Jian-xiong Cao, Lan-lan Tang

**Affiliations:** ^1^Graduate School, Hunan University of Chinese Medicine, Changsha, Hunan 410208, China; ^2^The First Hospital of Hunan University of Chinese Medicine, Changsha, Hunan 410021, China; ^3^School of Continuing Education, Hunan University of Chinese Medicine, Changsha, Hunan 410208, China; ^4^Ningxiang Hospital of Chinese Medicine, Ningxiang, Hunan 410399, China

## Abstract

Cancer is a complex disease with several distinct characteristics, referred to as “cancer markers” one of which is metabolic reprogramming, which is a common feature that drives cancer progression. Over the last ten years, researchers have focused on the reprogramming of glucose metabolism in cancer. In cancer, the oxidative phosphorylation metabolic pathway is converted into the glycolytic pathway in order to meet the growth requirements of cancer cells, thereby creating a microenvironment that promotes cancer progression. The precise mechanism of glucose metabolism in cancer cells is still unknown, but it is thought to involve the aberrant levels of metabolic enzymes, the influence of the tumor microenvironment (TME), and the activation of tumor-promoting signaling pathways. It is suggested that glucose metabolism is strongly linked to cancer progression because it provides energy to cancer cells and interferes with antitumor drug pharmacodynamics. Therefore, it is critical to unravel the mechanism of glucose metabolism in tumors in order to gain a better understanding of tumorigenesis and to lay the groundwork for future research into the identification of novel diagnostic markers and therapeutic targets for cancer treatment. Traditional Chinese Medicine (TCM) has the characteristics of multiple targets, multiple components, and less toxic side effects and has unique advantages in tumor treatment. In recent years, researchers have found that a variety of Chinese medicine monomers and compound recipes play an antitumor role by interfering with the reprogramming of tumor metabolism. The underlying mechanisms of metabolism reprogramming of tumor cells and the role of TCM in regulating glucose metabolism are reviewed in this study, so as to provide a new idea for antitumor research in Chinese medicine.

## 1. Introduction

**Figure 1:**
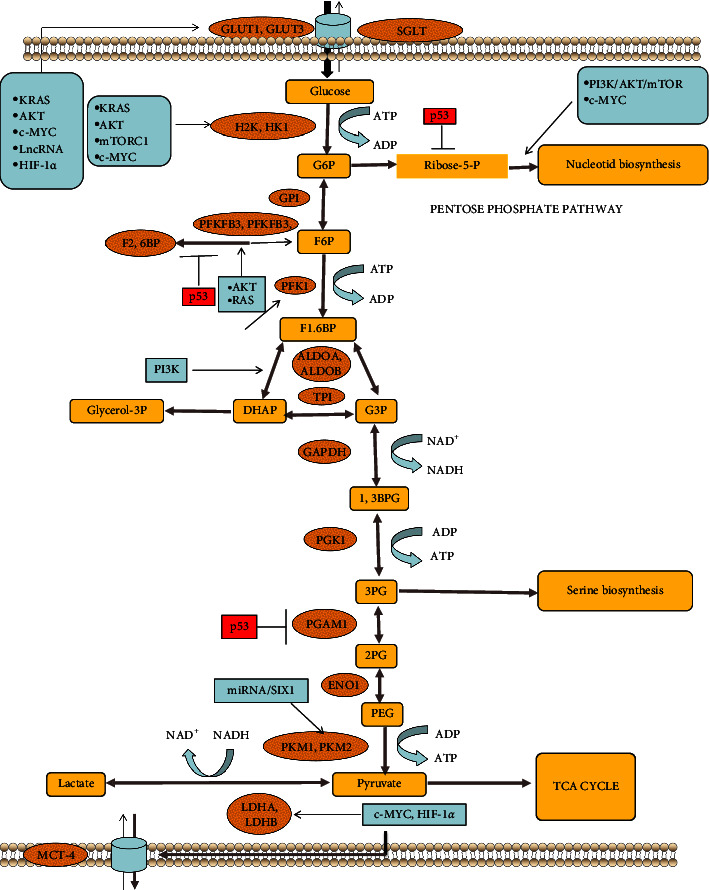
Reprogramming of glucose metabolism in cancer. Glucose metabolism-related enzymes and transporting proteins in cancer: the expression of GLUT1, GLUT2, SGLT, HK1, HK2, GPI, PFKFB3, PFKFB4, PFK1, ALDOA, ALDOB, TPI, GAPDH, PGK1, PGAM1, ENO1, PKM1, PKM2, LDHA, LDHB, and MCT4 are upregulated in the cancer glycolysis pathway. Different molecules affected in tumor cells like KRAS, AKT, c-MYC, LncRNA, HIF-1*α*, mTORC-1, and RAS promote, while p53 inhibits the expression of glycolytic proteins and enzymes which regulate cancer cell's glucose metabolism.

Cancer is a concerning health condition associated with high morbidity and mortality rates, and its prevalence is increasing globally. According to statistics, approximately 18.1 million new cancer cases and 9.6 million cancer-related deaths occurred in 2018. Therefore, it is critical to develop effective antitumor treatment modalities [1]. Otto Warburg et al. from Germany discovered in the early 1920s that the amount of glucose metabolized into lactate in cancer cells was 10 times higher than in normal tissues under aerobic conditions, implying that cancer cells preferred the glycolytic metabolic pathway over oxidative phosphorylation for energy production even in the absence of hypoxic conditions. This phenomenon, known as the “Warburg effect” or aerobic glycolysis, has since been confirmed by numerous studies [2, 3]. Specifically, the cancer cell mass may develop within the blood vessels first, and as it expands excessively away from the blood vessel, severe hypoxia may be induced. To adapt to such hypoxic conditions and promote cell proliferation, cancer cells increase glycolysis while suppressing mitochondrial oxidative phosphorylation and pyruvate metabolism, converting pyruvate in the cytoplasm into lactate, which is then exported from the cells [4]. Along with ATP production, the glycolytic pathway decomposes glucose into pyruvate. Furthermore, glycolysis may adapt to improve REDOX balance by increasing antioxidants and decreasing reactive oxygen species (ROS) [5]. Glucose can enter the PPP, which is an important step in cancer cell proliferation because it produces pentose phosphates (which aid in nucleic acid production), nicotinamide adenine dinucleotide phosphate (NADPH), ribose-5-phosphate, and nucleotide sugars, facilitating the production of various macromolecules and antioxidants, as well as the activation of protein glycosylation pathways [6]. Pyruvate, which is produced during glycolysis, is converted into alanine and lactate. Furthermore, this pyruvate could be converted into acetyl-CoA or oxaloacetate, which would then enter the tricarboxylic acid (TCA) cycle. This step is critical for the synthesis of ATP, fatty acids, and amino acids [7]. Therefore, Cancer cells exhibit distinct metabolic reprogramming when compared to normal cells, with atypical glucose metabolism frequently observed as the altered biochemical characteristics [8]. The modification of glucose metabolism is extremely important for tumor recurrence, metastasis, drug resistance, and poor cancer prognosis [9]. So far, the molecular mechanism underlying glucose metabolism reprogramming in cancer cells has remained largely unknown, and no universal mechanism underlying glucose metabolism reprogramming in diverse tumor cells has been identified. However, certain shared mechanisms for the regulation of glycolysis and glucose absorption across different tumor cells have been reported, allowing these cells to meet their anabolic requirements. Factors such as Hypoxia-Inducible Factor 1-Alpha (HIF-1*α*), tumor suppressor gene inactivation, oncogene activation, noncoding RNAs, glycolysis-associated transporters and enzymes, and the phosphatidylinositol 3-kinase (PI3K)-Akt-mechanistic target of rapamycin (mTOR) signal transduction pathway may lead to tumor cells preferring glycolysis over mitochondrial oxidation (Figure 1). Certain small molecules that target the glycolytic pathway have shown potent antitumor activity *in vitro* and *in vivo* [10]. Therefore, it is critical to understand the mechanism underlying the regulation of cancer cell deterioration by glucose metabolism, as well as the best way to exploit this reprogramming for efficiently targeting cancer cells [11]. In China, TCM has been widely used as a mainstream complementary and alternative therapy with distinct benefits for cancer patients [12]. TCM has been shown to promote anticancer therapeutic effects, reduce chemoradiotherapy toxicity, and improve patients' quality of life after surgery and in the late stages of cancer [13]. Although the antitumor mechanism of TCM is not fully understood, growing evidence suggests that TCM may exert its effects through glucose metabolism modulation [14]. In this context, the current study sought to investigate the mechanism underlying glucose metabolism in cancer, as well as the role of TCM in the regulation of glucose metabolism, in order to provide novel insights for improving tumor diagnosis, prognosis, and treatment by regulating the targets and pathways associated with glucose metabolism.

## 2. Reprogramming of Glucose Metabolism in Cancer

### 2.1. Reprogramming of the Enzymes and Transporters Associated with Glucose Metabolism in Cancer

Glycolysis is an important component of glucose metabolism and is linked to tumor development. To meet the energy, redox, and biosynthesis requirements for tumor development, tumor cells undergo reprogramming of metabolic enzymes and transport proteins associated with glucose metabolism in order to improve glucose absorption and speed up glycolysis and the production of metabolic end-products [15]. The first step in glycolysis is the transport of glucose to the cytoplasm across the plasma membrane with the help of glucose transporters (GLUTs). There are 14 known subtypes of human glucose transporters, of which GLUT3 and GLUT4 have been reported to have the highest affinity for glucose, whereas GLUT1 is commonly found to have an abnormal expression in various cancers, which has a significant impact on glucose metabolism in cancer [16, 17]. In addition to the GLUT protein family, sodium-dependent glucose transporters (SGLTs) are essential for glucose transport. In human cells, SGLTs exist in two isoforms: SGLT1, which has a low capacity but a high affinity for glucose, and SGLT2, which has a high capacity but a low affinity for glucose. Because SGLTs directly deliver glucose to cells, SGLT protein family-dependent cells always have a high glucose content, regardless of whether the transmembrane glucose content is high or low. It has been proposed that SGLT2 expression could aid in the detection of early lung adenocarcinoma and lung premalignancy. Therefore, SGLT2 could be used as a target in lung cancer (LC) to predict its early development and, ultimately, prolong patient survival [18]. SGLT2 is also expressed in prostate and pancreatic adenocarcinoma, and SGLT2 inhibitors have been shown to suppress glucose absorption and inhibit tumor cell proliferation in a pancreatic cancer model [19].

Certain critical glycolysis-associated enzymes are reportedly overexpressed in cancer cells and are strongly linked to cancer's poor prognosis. The hexokinase (HK) family catalyzes the first steps of glycolysis, where glucose is phosphorylated into glucose-6-phosphate (G-6-P). There are four mammalian isoforms of HK, namely, HK1-4, all of which are expressed at low levels in normal cells. In most cancers, HK1 and HK2 have the highest affinity for glucose transporters and are thought to play important roles in the regulation of antitumor efficacy and tumor prognosis [20]. It has been proposed that HK1 upregulation predicts poor prognosis in metastatic colorectal cancer (CRC) because it may suppress the miR-34a-5p, thereby inhibiting CRC cell proliferation and migration. Therefore, HK1 is regarded as a potential marker for predicting the prognosis of metastatic CRC [21]. Because HK2 overexpression has been found in a variety of cancers, it could be used as a therapeutic target in cancer treatment. For example, HK2 is thought to be involved in tumorigenesis and glycolysis in hepatocellular carcinoma (HCC) cells, and increased HK2 expression in HCC patients is associated with lower overall survival (OS), whereas inhibiting HK2 activity reportedly promoted the therapeutic effect of sorafenib in an *in vivo* model of HCC. Furthermore, inhibiting HK2 may alter the metabolic profiles of cervical cancer (CC) cells by decreasing their reliance on glycolysis while increasing mitochondrial activity and increasing the radiosensitivity of HPV (+) CC cells [22, 23].

One of the housekeeping cytosolic enzymes, glucose-6-phosphate isomerase (GPI), catalyzes the mutual conversion of G6P and fructose-6-phosphate (F-6-P), which is essential for the gluconeogenic and glycolytic pathways. According to one study, GPI mRNA expression is a promising biomarker for the prognosis of gastric cancer (GC) [24]. The rate-limiting step in the glycolytic pathway is catalyzed by the phosphofructokinase (PFK) enzyme, which converts F6P to fructose-2,6-biphosphate (F-2, 6B-P) and fructose-1,6-biphosphate (F-1, 6B-P). The two steps of this phosphorylation consume two molecules of ATP. Therefore, PFK has two isoforms: PFK-1 and PFK-2. The PFK-1 isoform, which includes PFK-platelet (PFKP), PFK-muscle (PFKM), and PFK-liver (PFKL), is expressed differentially in human tissues and organs [25]. Overexpression of PFK-1L and/or PFK-1M promotes increased flux through the glycolytic pathway (to generate H^+^ and ATP) as well as an increase in intermediate metabolites for biosynthesis [26]. PFKP has been found to be overexpressed in a variety of cancers, including breast cancer (BC) and glioblastoma (GBM) [27]. According to one study, PFKP plays a critical role in *β*-catenin transactivation mediated by epidermal growth factor receptor (EGFR) activation, which may enhance brain tumor development and GBM cell proliferation, invasion, and migration, highlighting the possibility of targeting PFKP Y64 phosphorylation to treat GBM [28]. Therefore, PFKP, rather than PFKM and PFKL, is thought to be strongly linked to cancer growth, survival, and metastasis, and could be used as a marker for poor prognosis in cancer [29]. Inhibiting the glycosylation of PFK-1 may thus reduce cancer cell proliferation [30].

The PFK-2 family contains several isozymes, namely, positive PFKFB1-4, which are encoded by four different genes. Typically, the cancer-specific isoenzymes PFKFB3 and PFKFB4 keep the F-2,6-BP content in the cytoplasm stable. Targeting PFKFB3 and PFKFB4 in tumor cells has been shown in a few in vitro studies to inhibit glycolysis, thereby slowing tumor development. Furthermore, the association of PFKFB3 expression with PFKFB4 level determines the significance of PFK-2 in predicting the prognosis of neuroblastoma, indicating the need to further investigate the effects of these two PFK-2 isoenzymes. Notably, the expression levels of these two PFK-2 isoenzymes have been shown to predict the prognosis of certain cancers [31, 32]. PFK-2 expression has been found to be elevated in a variety of invasive primary tumors, including CRC, BC, and CC. Furthermore, studies have shown that the PFK inhibition test can detect tumors in the clinic [33, 34].

Later, members of the aldolase (ALDO) family, specifically isozymes A, B, and C, which are encoded by three different genes and exhibit differential expression in certain cancers), are capable of degrading fructose-1,6-biphosphate to generate dihydroxyacetone phosphate (DHAP) and glyceraldehyde-3-phosphate (G-3-P) [35, 36]. It was recently proposed that abnormally expressed ALDOA aided the development of CRC, GC, CC, LC, and HCC by increasing drug resistance, tumor cell migration and proliferation, and epithelial-mesenchymal transition (EMT) [37–41]. Overexpression of ALDOB is associated with the advanced rectal cancer (RC) stage and the induction of chemotherapy-related adverse effects in patients with RC, and it could thus serve as a novel prognostic biomarker for RC [42]. ALDOB downregulation, on the other hand, is associated with multiple malignant characteristics of HCC, partly through TET1 expression, and could thus be considered a prognostic biomarker for hepatocellular carcinoma, particularly at the early stage of disease [43]. Furthermore, ALDOC levels are linked to oral squamous cell carcinoma (OSCC) and may thus serve as a candidate biomarker in OSCC, in addition to allowing for the modulation of OSCC development [44].

GAPDH was initially thought to have stable expression and was thus frequently used as a reference gene. However, new evidence suggests that GAPDH may be overexpressed in cancer to boost glycolysis and accelerate tumor growth [45]. During the payoff stage of glycolysis, GAPDH oxidizes and phosphorylates G3P to produce 1,3-biphosphoglycerate (1,3-BPG). Furthermore, GAPDH influences glycolysis by modulating metabolic pathways such as the mammalian target of the AMPK signal transduction pathway [46].

Phosphoglycerate kinase (PGK) catalyzes the reversible ATP production reaction in glycolysis by converting 1,3-BPG to 3-phosphoglycerate (3-PG). Humans have two PGK isoenzymes, PGK1 and PGK2, with the former being overexpressed in various cancers and modulated through a variety of mechanisms. In pancreatic cancer (PAC), for example, the transcription factor nuclear factor of activated T-cells 5 facilitates the rewiring of the glycolytic phenotype and PAC growth via PGK1 expression in HCC cells and metastatic GC cells [47, 48]. According to Li et al. and colleagues, oncogenic mutations or hypoxia that activated the extracellular regulated protein kinases (ERK) signal transduction resulted in the mitochondrial translocation of PGK1, which resulted in the activation and phosphorylation of PDHK1 for the promotion of the Warburg effect and suppression of pyruvate metabolism in mitochondria, ultimately accelerating cancer cell growth. Macrophages, the most common leukocytes in the TME, may also hasten cancer development [49]. According to Zhang et al. and colleagues, macrophages in resident tissues may produce IL-6 to regulate the PGK1-catalyzed reaction in cancer cells by promoting PGK1 phosphorylation dependent on PDPK1, thereby enhancing cancer cell glycolysis and tumorigenesis [50].

Phosphoglycerate mutase 1 (PGAM1), an enzyme that catalyzes the mutual conversion of 2-phosphoglycerate and 3-phosphoglycerate during glycolysis, is commonly upregulated in various human cancers and plays an important role in regulating anabolic activity and glycolysis to promote tumor development [51]. Upregulation of PGAM1 has been reported in CRC, BC, LC, and HCC [52]. The stable silencing of PGAM1 in PAC resulted in significantly reduced lactate generation, glycolytic rate, lipogenesis, RNA production, cell proliferation, and oxidative PPP flux, as recently reported by Zhao et al. and colleagues [53].

ENO1, the enzyme that converts 2-phosphoglycerate to phosphoenolpyruvate, on the other hand, is essential for aerobic glycolysis and speeds up cancer development. Furthermore, by regulating the miR-22-3p-ENO1 axis, circ-ENO1 has been shown to promote glycolysis and the development of lung adenocarcinoma [54].

Pyruvate kinase (PK) is a glycolytic enzyme that catalyzes the conversion of phosphoenolpyruvate to pyruvate as well as the production of ATP molecules during glycolysis. PK has four isozymes, L, R, M1, and M2, which are encoded by the PKM and PKL genes. PKM1 is the primary isoenzyme in normal tissues; when normal tissues become cancerous, PKM1 decreases and PKM2 takes its place in glucose metabolism [55]. Furthermore, PKM1 is transformed into PKM2 during tumor development, and backward homologous conversion of PKM2 into PKM1 suppressed aerobic glycolysis and delayed cancer progression in the xenograft nude mouse model [56]. PKM2, an enzyme in the cell glycolytic pathway, increases the glycolysis rate to provide energy to tumor cells and either produce lactic acid via its lactate dehydrogenase activity or metabolizes to acetyl-CoA via its pyruvate dehydrogenase activity. The acidic nature of the TME promotes tumor growth [57]. The inactive dimeric form and the active tetrameric form of PKM2 are thought to play important roles in aerobic glycolysis and tumor cell growth [58]. Furthermore, the involvement of PKM2 in glycolysis and glutamine decomposition regulation contributes to the transformation of cancer cell metabolism [59]. PKM2 is an active PK that participates in glycolysis to meet the needs of cancer cells. Furthermore, it functions as an inactive protein kinase dimer that modulates the biosynthetic metabolism of cancer cells, which is required for the process of growth stimulation [60, 61]. Meanwhile, PKM2 functions as a nuclear co-transcription factor, modulating gene transcription. PKM2, for example, suppresses the PI3K-AKT signal transduction pathway to promote cell migration as well as autophagy, which promotes GC occurrence [62]. Meanwhile, PKM2 overexpression in intrahepatic cholangiocarcinoma promotes cell proliferation, invasion, and migration via oncogene regulation and activation of the PI3K-AKT-mTOR signal transduction pathway [63]. PKM2 overexpression promotes the proliferation and growth of ovarian cancer (OC) cells by increasing the expression of recombinant cyclin D1 (CCND1) [64]. Under hypoxic conditions, circMAT2B increases glycolysis via the circMAT2B/miR-338-3p/PKM2 axis, promoting the progression of hepatocellular carcinoma [65]. Therefore, PKM2 plays an important role in cancer development via a variety of pathways, including glucose metabolism, nuclear signal transduction, protein synthesis, and protein interaction [66].

Lactate dehydrogenase (LDH) is a key enzyme that converts pyruvate to lactate under hypoxic conditions, generating the nicotinamide adenine dinucleotide (NAD+) stock required to keep the glycolytic flux going [67]. The LDHB-encoded H and LDHA-encoded M subunits make up the homo- and hetero-tetramers of LDHs. Because of the high concentration of lactic acid, LDH enzymes with higher M-subunit contents (LDHA proteins) are abnormally overexpressed in a variety of cancers, increasing lactic acid levels and promoting tumor metastasis and invasion, as well as mediating tumor immune escape [68]. As a result of its close relationship with tumor metastasis, tumor staging, tumor recurrence, treatment resistance, and patient survival, LDHA could serve as an effective tumor diagnosis and prognosis factor [69].

### 2.2. Reprogramming of the TME

Recent evidence suggests that tumor cell energy metabolic interactions in the TME either support tumor growth, metabolism, and maintenance or contribute to antitumor immunity impairment. Therefore, understanding glucose metabolism in the TME is critical for guiding the development of novel drug targets and cancer treatment formulation. The TME is made up of cancer-associated fibroblasts (CAFs), tumor-associated macrophages (TAMs), and a slew of cancer cells, stromal cells, and immune cells. TME puts tumor cells through a slew of tests, including oxidative stress, physical pressure, hypoxia, nutrient competition and deprivation, and immune surveillance [70, 71]. The conditions in the TME have a significant impact on cancer cell glucose metabolism, and the hypoxic microenvironment shaped by tumors prevents tumor cells from carrying out oxidative phosphorylation or other oxygen-requiring reactions while disrupting the redox balance, affecting transcription and cell signal transduction [72]. Increased glycolytic carbon flux may result in excessive lactic acid production, acidifying the microenvironment and contributing to the progression and metastasis of certain cancers [73]. TME components may influence tumor glucose metabolism by regulating signaling pathways. Finally, reciprocal interactions between tumor cells and their surrounding microenvironment impose a selective pressure that shapes glucose metabolism in tumor cells, allowing them to become more aggressive [71]. As a result of the glucose metabolic interactions with the oxidative cells, tumor cells develop rapidly within the TME. Knowledge of the specific TME components involved in cancer cell glucose metabolism and the underlying mechanism by which the TME targets glucose metabolism would aid in the development of more effective anticancer therapeutic strategies. The modulation of cancer cell glucose metabolism by immunocytes and fibroblasts in the TME will be discussed further in this context.

In several solid tumors, CAFs are the primary cellular component of the TME [74]. CAFs differ from normal fibroblasts in several ways. CAFs, for example, are larger spindle mesenchymal cells that are linked to cancer cell proliferation, invasion, and migration [75]. Furthermore, CAF-derived exosomes are thought to suppress oxidative phosphorylation in mitochondria, promoting glycolysis and glutamine-mediated reductive carboxylation in cancer cells [76, 77]. Therefore, glucose is converted into lactate, resulting in low pH conditions to regulate TME. Furthermore, the glycolysis metabolites pyruvate and lactate are absorbed into the surrounding cancer cells for use in the TCA cycle, increasing the efficiency of energy generation (ATP produced by oxidative phosphorylation) and promoting cell proliferation. The “Reverse Warburg Effect” refers to this phenomenon [78]. CAFs have recently been observed to initiate metabolic reprogramming in the TME to support cancer cell proliferation and metastasis [79, 80]. Hypoxia and oxidative stress have been shown to effectively activate the glycolysis flux in CAFs via HIF1-*α*. Cav-1 knockout fibroblasts could stabilize HIF-1*α* under normal oxygen conditions by increasing reactive oxygen species in cells [81]. On the one hand, HIF1-*α* promotes glycolysis by regulating glucose transporters, glycolytic enzymes, and the genes that encode them [82]. Furthermore, HIF-1*α* may influence CAF secretion. Sun et al. [83] discovered that the levels of hypoxia-mediated oxidized ataxia-telangiectasia mutated were closely related to the glycolytic activity. Furthermore, hypoxic ATM activation and oxidation promoted the upregulation of PKM2 and the phosphorylation of GLUT1, facilitating lactate synthesis. Furthermore, lactate is the metabolism-mediating factor that couples Monocarboxylate transporter4 (MCT4)-derived CAFs with cancer cells (absorbs by MCT1), promoting cancer cell invasion and enhancing mitochondrial activity [84, 85]. CAFs-produced lactate may be used by cancer cells as an additional nutrient source in addition to its effect on immune response regulation [86].

Several tumor cells have been observed to use glycolytic metabolism to produce lactate and acidify the TME, despite the fact that this has a significant impact on T cell-regulated anticancer immune reactions and tumor-infiltrating myeloid cell activities [87]. Cancer cell metabolic reprogramming causes immunosuppression, which is important in tumor development, drug resistance, and treatment failure [88]. On the one hand, cancer cells have a high energy demand, resulting in a severe energy shortage, making the TME a demanding metabolic environment and creating fierce competition for glucose, reducing glucose availability to T cells. On the other hand, high glycolysis rates in cancer reduce immunostimulatory signals, resulting in a variety of effects on T cell function and antitumor immune responses [89, 90]. Dendritic cells (DCs) are associated with anticancer activity and play an important role in initiating the anticancer T-cell response. Lactic acid accumulation within the TME may alter the functions and antigen phenotype of these DCs, and lactic acid alone or in combination with IL-6 and macrophage colony-stimulating factor 1 (M-CSF) may result in a tumor-associated DC phenotype [91]. Macrophages in tumor tissues (tumor-associated macrophages), like those in normal tissues and organs, play an important homeostatic role and are linked to tumor proliferation, metastasis, and invasion [92]. Glucose deficiency or lactic acid accumulation within the TME destroys the metabolic programs and signaling cascades that support macrophage proinflammatory polarization, promoting the development of M2-like macrophages. Furthermore, the use of aerobic glycolysis may convert M2 tumor-associated macrophages into M1 tumor-associated macrophages, which suppress tumor development [21]. Another study found that lactate, as mediated by HIF-1*α*, could induce TAM polarization, which is characterized by VEGF expression and differentiation into M2-like macrophages [93]. Furthermore, glycolysis in tumors increases the expression of granulocyte-macrophagecolony-stimulating factor (GM-CSF) and granulocyte colony-stimulating factor (G-CSF), facilitating the recruitment of myeloid-derived suppressor cells (MDSCs) into the TME and influencing T cell-mediated tumor immunity and tumor development [94]. Husain et al. and colleagues demonstrated that cancer-produced lactate inhibited natural killer (NK) cell activity by directly downregulating NKp46as and indirectly upregulating MDSCs to suppress NK cytotoxicity [95]. Tumor cells control T cell metabolism by competing for glucose and, as a result, inhibiting T cell mTOR activity, glycolysis ability, and IFN-*γ* production in order to achieve immune escape. Furthermore, hypoxia plays an important role in T cell production and activity and has been shown in animal and cell models to suppress T-cell growth. T cell infiltration was increased, pro-inflammatory factors were upregulated, tumor proliferation was inhibited, and mouse survival was improved after oxygen supplementation in mice, which alleviated hypoxia in the microenvironment [96]. Therefore, the hypoxic and acidic microenvironment induced by tumor cell glycolysis may result in metabolism-mediated T cell dysfunction, which could be one of several mechanisms by which tumor cell glycolysis mediates immune escape. The interaction of the programmed cell death protein 1 (PD-1) and its ligand (PD-L1 or PD-L2) may increase aerobic glycolysis in tumor cells by suppressing the PI3K-AKT-mTOR signal transduction pathway, thereby inhibiting T-cell activation while promoting immune escape [97, 98].

### 2.3. The Regulation Mechanism of Tumor Glucose Metabolic Reprogramming

It is becoming clear that coordinated networks of signaling pathways control the reprogramming of glucose metabolism in cancer cells to promote tumor growth and stress resistance. There are numerous regulating factors in cancer cells that promote glucose metabolism, including protooncogenes (c-Myc), tumor suppressors (p53), transcription factors (HIF-1*α*), and signal transduction pathways (PI3K-Akt-mTOR). Tumor suppressors and oncogenes control the promotion of aerobic glycolysis for the generation of ATP (the Warburg effect), lactate, and pentose phosphate pathway (PPP), thereby promoting cancer cell growth and proliferation. Oncogene activation generally enhances the pentose phosphate pathway and the glycolytic pathways in cancer cells, as well as promoting the malignant phenotype and demonstrating high expression in various cancer cells, whereas tumor suppressor gene inactivation reverses the Warburg effect to negatively influence the oncogenic metabolic adaptation of cancer cells [99]. Besides, increasing evidence suggests that noncoding RNAs, especially micro-RNAs (miRNAs) and long noncoding RNAs (lncRNAs) via glucose consumption and trafficking, are involved in cancer cell proliferation, differentiation, metastasis, and apoptosis. Alternatively, noncoding RNAs may have an indirect effect on cancer-related signal transduction pathways [100].

#### 2.3.1. c-Myc

The oncogene Myc encodes the transcription factor c-Myc, which is involved in the control of cell proliferation and metabolism and is upregulated in various cancers such as BC, bladder cancer (BCA), PAC, and CRC [101]. According to reports, c-Myc has been linked to an increase in mitochondrial ROS, which leads to mitochondrial dysfunction and cancer cells switching to glycolysis for energy production [102]. Simultaneously, c-Myc promotes glycolysis, glucose absorption, and lactate synthesis in cancer cells, which aids in metabolic reprogramming. The activation of several genes associated with glycolytic enzymes (such as HK2, GAPDH, ENO1) and glucose transporters (such as SLC2A1, SLC2A2, and SLC2A4) by c-Myc results in increased glycolysis. Furthermore, NDRG2 mediated by c-Myc has been shown to suppress glutamine synthesis and glycolysis, thereby significantly suppressing CRC proliferation [103].

#### 2.3.2. p53

Several tumor suppressors, such as p53, influence glucose metabolism in cancer cells by modulating the switch between glycolysis and OXPHOS, preventing the development of more aggressive tumor phenotypes via various pathways such as the PI3K-Akt pathways [104]. It has been reported that p53 promotes oxidative phosphorylation by regulating its downstream genes TP53-induced glycolysis regulator, which is a F-2,6-BP inhibitor (120), while decreasing glycolysis by downregulating GLUT1/4 and GLUT3 (by inhibiting the NF-*κ*B factor) and hexokinase 2 (HK2) and participating in the phosphoglycerate mutase (PGM) degradation routes [105, 106]. Furthermore, p53 may improve mitochondrial oxidation by activating the respiratory chain's SCO_2_ gene. Furthermore, p53 mutation in human cancers is frequently associated with AMP-activated protein kinase (AMPK), the main sensor of cellular energy, resulting in increased aerobic glycolysis [107].

#### 2.3.3. HIF-1*α*

Hypoxia (decreased oxygen condition) can stimulate increased glucose consumption and lactate production in the cells. This process is regulated by HIF-1*α*, which is a transcription factor complex. HIF-1*α*, in collaboration with c-Myc, promotes glucose uptake by cells and accelerates tumor cell aerobic glycolysis by increasing the expression of GLUTs (primarily GLUT1 and GLUT3) or additional glycolytic enzymes that rely on HIF-1*α* (such as LDHA, MCT4, and GAPDH) [108, 109]. As confirmed in the PCA experiment, HIF-1*α* is activated in high glucose metabolic samples both in vitro and in vivo, which is closely related to the invasion and metastasis capacity of PCA [110].

#### 2.3.4. PI3K-Akt-mTOR

Because of its importance in coordinating cell biosynthesis and aerobic glycolysis in cancer cells, the PI3K-Akt-mTOR signal transduction pathway is also considered a candidate target pathway in anticancer therapy [111]. Through the upregulation of GLUTs, PKM2, and HK2, the PI3K-Akt pathway promotes cancer development by facilitating certain cell events such as glycolysis. Firstly, PI3K-Akt promotes glucose uptake in cells by increasing the membrane translocation and expression of GLUT4 [112]. In addition, PI3K-Akt promotes glycolysis by activating HK and by the binding of HK2 to the voltage-dependent anion channel in mitochondria [113]. Moreover, PI3K-Akt could regulate glycolytic enzymes indirectly by regulating the expression of AMPK and HIF-1*α* [114].

#### 2.3.5. Noncoding RNAs

Many noncoding RNAs, such as miRNAs and lncRNAs, play an important role in mediating tumor glucose metabolism. miRNAs are noncoding single-stranded RNA molecules of about 20–24 nucleotides in length encoded by endogenous genes and are involved in the regulation of post-transcriptional gene expression. miRNAs play an important role in the occurrence and development of tumors. Studies have shown that downregulation of miR-214 can inhibit lactate production, glucose consumption, and cell proliferation, and regulate the PTEN/AKT/mTOR pathway by targeting PTEN [115]. An experiment indicated that miR-98 was significantly down-regulated in CRC tissues and suppressed the Warburg effect by targeting HK2 [116]. SIX1 has been shown to increase cancer cell proliferation and glycolysis by modulating PKM2 expression. MiRNAs that function as oncogenes or tumor suppressors may target SIX1 to promote or suppress cancer development and are thus linked to cancer cell metabolism and apoptosis. Therefore, the miRNA/SIX1 axis may be targeted in anticancer therapy [117, 118]. MiR-340, which increases glucose uptake and lactate secretion by increasing the expression of GLUT1, was decreased in oral squamous cell carcinoma [119]. MiR-34a was reported to regulate key enzymes including HK1, HK2, GPI, LDHA, and PDK1 [120]. MiR-210-3p facilitated aerobic glycolysis by modulating the downstream glycolytic genes of HIF-1*α* and p53 in triple-negative breast cancer (TNBC) [121]. lncRNAs are a class of noncoding single-stranded RNA molecules with a length of more than 200 amino acids, which have important functions in transcription, silencing, activation, chromosome modification, and nuclear transport. Increasing evidence suggests that lncRNAs have the potential to alter glucose metabolism, primarily by effectively regulating critical glycolytic enzymes or cancer-associated signal transduction pathways such as the p53 and PI3K-Akt-mTOR pathways [122]. Plasma lipid-associated lncRNAs have been shown to be capable of regulating the stabilization of the normoxic HIF-1*α* [123]. Miah et al. found that the lncRNA promotes TNBC tumorigenesis by regulating the HIF-1*α*-associated signaling pathway [124–126].

## 3. Therapeutic Intervention with TCM in relation to Glucose Metabolic Reprogramming

Given the limited efficacy of single-target inhibitors of the glucose metabolism reprogramming against complex cancers and the need for safe anticancer agents, TCM with multiple targets and antitumor effects holds tremendous potential. Related research found that TCM effectively inhibited the malignant proliferation of tumor cells by suppressing the expressions of transporters and enzymes associated with glucose metabolism (such as GLUT, MCT, PFK, PKM, HK, LDH, and ENO), modulating mutations in associated oncogenes (such as p53), and suppressing the aberrant activation of a signaling pathway. Therefore, this part aims to present an overview of the TCM which demonstrated, an effective anticancer activity by targeting the glycolytic proteins, enzymes, and related regulatory signaling pathways [127].  Table 1 contains detailed information about these treatments:

  Chrysin: Xu et al. demonstrated that chrysin, a bioactive flavone derived from blue passionflower (*Passiflora caerulea*), inhibited glycolysis by reducing the expression of HK2 in HCC [128].
  Kaempferol: a study investigated the capacity of kaempferol to block glucose uptake in BRAC. The resulted indicated that kaempferol inhibited glucose uptake with a 40% decrease in GLUT1 mRNA levels [129].
  Biochanin, one of the predominant isoflavones in *Trifolium pratense*, suppressed CRC cell and CC cell proliferation and induce apoptosis via restricting glycolysis and mitochondrial OXPHOS. The identified mechanism implied the phosphorylation of AKT and mTOR, along with decreased expression of HIF-1*α*, GLUT1, HK2, and LDHA. Moreover, similar results were observed in glioma cells [130, 174].
  Quercetin (QUE), which has diverse effects including antioxidant, anti-inflammatory, vasodilatory, and anticancer effects, is a natural multifunctional flavonoid [131]. Hamilton et al.reported that quercetin was shown to bind to an exofacial site of GLUT1 without being transported into cells [132]. Jia et al. found that quercetin could reduce the protein levels of PKM2 and GLUT1 in BC. Besides, QUE was proved to be able to decrease glycolytic metabolism and cause growth suppression in BC through Akt-mTORpathway-mediated autophagy induction [133]. Novel evidence has shown that QUE can inhibit the proliferation of glycolysis-addicted HCC cells by reducing HK2 and Akt-mTOR pathways [134].
 
*α*-Hederin, a potent bioactive compound of *Pulsatilla chinensis* (Bunge) Regel (Ranunculaceae), inhibits the growth of LC A549 cells *in vitro* and *in vivo* by decreasing SIRT6-dependent glycolysis [135].
  Worenine: in a study designed to explore its mechanisms against CRC, worenine, an isoquinoline alkaloid isolated from *Coptis chinensis*, significantly decreased the protein and mRNA levels of PFKL and promoted glucose accumulation by negatively regulating HIF-1*α* [136].
  Curcumin: Wang et al. found that curcumin, the main active component of turmeric, inhibited aerobic glycolysis in CRC and induced mitochondrial-mediated apoptosis through HK2 in an Akt-dependent manner [137]. According to another study, curcumin triggered downregulation of PKM2 via mTOR-HIF-1*α* inhibition in non-small-cell lung cancer (NSCLC), breast adenocarcinoma (BRCA), cervical adenocarcinoma (CAC), and PAC, reversing the Warburg effect [138].
  Bergapten, a derivative of psoralen found in bergamot essential oil, was shown to inhibit BC cell growth by directly decreasing PFK-1 expression and lactate production rates [139].
  Triterpenoids: according to network analysis and experimental validation, triterpenoids isolated from *Schisandra chinensis* have anticancer activity by suppressing cancer cell glycolysis. Furthermore, these molecules could reduce the expression of ENO1, PFKFB3, and ALDOA in the CRC in a dose-dependent manner [140].
  Apigenin: experiments have shown that the mechanism underlying apigenin's (an abundantly present flavonoid) anticancer effect is the inhibition of GLUT1, HK2, and PKM2, which significantly reduces tumor cell glycolysis via the HIF-1*α* and PI3K-Akt-mTOR signaling pathway [141–143].
  Shikonin effectively inhibited the progression of Lewis lung cancer and B16 melanoma in a dose-dependent manner, with the main mechanism involving the inhibition of cancer cell glycolysis by reducing the phosphorylation level of PKM2, resulting in a decrease in ATP levels [144].
 
*Carpesium abrotanoides* (L.) root: Chai et al. identified *Carpesium abrotanoides* (L.) root as a source of natural compounds targeting glucose metabolism. The study results indicated that *Carpesium abrotanoides* (L.) root significantly inhibited the glycolytic flux in BRCA cells and melanoma (MEL) cells. The mechanism involves the downregulation of PKM2, MCT4, and GLUT1 expression [145].
  Nobiletin was found to inhibit the growth of OSCC CAL-27 and TCA-8113 cells *in vitro* by causing G1 cell cycle arrest. Nobiletin also reduced the levels of PGK during glycolysis and induced mitochondrial dysfunction, indicating that it could be useful in antitumor treatment and drug resistance reduction [14].
  Epigallocatechin was found as potent and specific LDH inhibitors in silico ligand binding virtual screening and in vitro enzymatic activity assay by Martin et al. [146].
  Wogonin, a flavonoid extracted from *Scutellaria baicalensis* Georgi, can regulate p53 downstream glycolytic factors, such as GLUT1, by decreasing its protein and mRNA level in CRC, ovarian cancer (OC), and HCC [147]. Moreover, an experiment exhibited that wogonin decreased the expression of glycolysis-related proteins (HK2, PDHK1, LDHA), glucose uptake, and lactate generation by inhibiting HIF-1*α* and PI3K-Akt signaling pathway in CRC [148].
  Scutellarin: of the glycosyloxyflavone class, scutellarin has proved to have potent inhibitory effects against PKM2. In an in vitro study, quercetin was shown to inhibit the proliferation of cervical cancer cells by inhibiting the activity of PKM2 [149]. In a more recent study, inhibition of PKM2 by scutellarin led to the resensitization of oxaliplatin-resistant CRC cells to oxaliplatin treatment [150].
  Matrine has been shown in studies to significantly inhibit the expression of GLUT1, HK2, and LDHA, which are downstream targets of hypoxia-induciblefactor-1*α*, which regulates glucose metabolism, thereby inhibiting the development of colon cancer cells. Therefore, matrine was proposed as an antitumor drug for the treatment of colon cancer that targets the glucose metabolism mediated by HIF-1*α* [151]. In addition, matrine and the HK2 inhibitor lonidamin promote human myeloid leukemia (ML) cell apoptosis in a synergistic effect through the Warburg effect mediated by HK2 [152].
  Costunolide, a sesquiterpene lactone first isolated from costus (*Saussurea lappa* Clarke), was proved to decrease glycolysis-associated activation of hepatic stellate cells via HK2 inhibition [153].
  Spatholobus suberectus, the active ingredient of *S. chinensis*, inhibits the growth of BC cells by reducing the expression of LDHA [154].
  Oleanolic acid (a triterpenoid widely studied) suppressed the expression of PKM2 in glioma cells in a dose/time-dependent manner, thereby exerting an inhibitory effect on aerobic glycolysis in Malignant Glioma [155]. Additionally, Oleanolic acid also can inhibit HIF-1*α*-mediated glycolysis in GC [156].
  Silybin: an experiment found that Silybin, a flavonoid extracted from Silybum marianum, inhibited the growth of doxorubicin-resistant CRC cells through direct competitive inhibition of GLUT4-mediated transport [157].
  Prosapogenin A: in CC, HCC, and BRAC, Prosapogenin A (a saponin extracted from Veratrum sp.) inhibited cell proliferation and promoted apoptosis, and its mechanism is related to the downregulation of the expressions of glycolysis-related genes, STAT3, GLUT1, HK, and PFKL [158].
  Resveratrol: Zambrano et al. introduced comprehensively resveratrol's (a widely studied polyphenol) inhibitory effects on glucose uptake [159]. In MCF7 BC, resveratrol decreased glucose consumption and ATP content, effects which were directly correlated with PFK-1 inhibition. A study has reported that resveratrol suppresses cancer cell glucose uptake, and its mechanism is related to the inhibition of the accumulation of HIF-1*α* and the expression of GLUT1 [160]. Moreover, resveratrol was shown to directly inhibit the proliferation of ovarian cancer cells and NSCLC via impairing glycolysis and targeting the Akt signaling pathway [161, 162].
  Deguelin, a naturally occurring flavonoid from *Mundulea sericea*, was reported to reduce the viability and activation of NSCLC by downregulating HK2 expression and blocking Akt phosphorylation [163].
  Ginsenoside 20 (S)-Rg3: studies have shown that Ginsenoside 20 (S)-Rg3, the main antitumor bioactive component of ginseng, effectively inhibited the Warburg effect through STAT3 pathways in OC [164].
  Betulinic acid significantly reduced the expression of key LDHA and PDK1 and the production of lactic acid in BC, and lead to the transformation of the cellular energy phenotype into a quiescent state [165].
  Tanshinone IIA: studies have confirmed that Tanshinone IIA inhibits glucose metabolism leading to apoptosis in CC by downregulating the expressions of GLUT1, PKM2, and HK2 [166].
  Realgar inhibits glucose metabolic reprogramming and suppresses LC cell growth in vivo experiments via inhibiting the activation of HIF-1*α* and PI3K-Akt-mTOR signaling [167].
  Huaier: in vivo and in vitro experiments suggested that Huaier suppressed glycolysis, glucose transport, and lactic acid accumulation and inhibited LC cell growth possibly through the PI3K-AKT-HIF-1*α* pathway [168].
  Cardamonin: a study found that cardamonin inhibited the growth of the TNBC cell line MDA-MB-231 by suppressing HIF-1*α* mediated glycolysis [169].
  Coptisine: in vitro experiments demonstrated that coptisine blocks the secretion of exosomal circCCT3 from cancer-associated fibroblasts to reprogram glucose metabolism in HCC [170].
  Ginsenoside compound K is reported to suppress LC cell growth via HIF-1*α* mediated metabolic alteration, contributing to novel anticancer therapy by targeting glucose metabolism [171].
  Atractylide I: experiments proved that atractylide I inhibited the growth of CRC cells and changed their glucose metabolism via AKT-mTOR signaling [172].
  Licochalcone A: Wu et al. discovered that Licochalcone A, a chalcone derived from licorice, significantly reduced glucose consumption and lactic acid generation in GC by inhibiting the Akt/HK2 pathway, indicating that Licochalcone A inhibits GC cells growth and induces apoptosis [173]. Similarly, in BRAC, licochalcone A triggered glycolysis inhibition and suppressed PI3K/Akt/mTOR activation, promoting autophagy and apoptosis [175].

## 4. Conclusion and Future Prospects

Cancer progression is aided by the reprogramming of glucose metabolism in cancer cells. However, current knowledge of glucose metabolism in cancer is limited and has yet to be fully translated into clinically useful applications. It is likely that the high, albeit inefficient, rate of ATP production in cancer cells is not their primary selective advantage and that these cells benefit more from the high levels of intermediate products diverted to pathways that generate nucleotides, amino acids, lipids, and NADPH. The process of glucose metabolism in cancer and its underlying mechanism may be regulated by a variety of associated enzymes, oncogenes, tumor suppressors, and noncoding RNAs. The discovery of the critical node (s) in the network of pathways that regulate glucose metabolism may lead to the discovery of new targets for antitumor therapies. However, given the complexity of the process of glucose metabolism reprogramming in cancer, it is assumed that anticancer therapy focusing on glucose metabolism will be limited, and inhibitors focusing on a single glucose metabolism modulator will be ineffective in treating cancer. Therefore, more emphasis should be placed on developing combination drug therapies for cancer treatment. TCM is widely accepted in China for its “multicomponents and multitargets” manifestations. Moreover, TCM may be effective in preventing and treating cancer by directly targeting glycolytic enzymes or indirectly targeting glycolytic pathways. It is an important adjuvant therapy for cancer and can be the focus of future research.

## Figures and Tables

**Table 1:** The treatment of TCM on reprogramming of glucose metabolism in cancer.

TCM	Targets	Cancer	Reference
Chrysin	HK2	HCC	[127]
Kaempferol	GLUT1	BRCA	[128]
Biochanin	HIF-1*α*, GLUT1, HK2, LDHA, and AKT-mTOR	CRC, CC, and glioma	[129, 130]
Quercetin	PKM2, GLUT1, HK2, and Akt-mTOR	BC and HCC	[131–133]
*α*-Hederin	SIRT6	LC	[134]
Worenine	PFKL and HIF-1*α*	CRC	[135]
Curcumin	HK2, PKM2, and mTOR-HIF-1*α*	CRC, NSCLC, BRAC, CC, and PAC	[136, 137]
Bergapten	PFK-1	BC	[138]
Triterpenoids (*Schisandra chinensis*)	ENO1, PFKFB3, and ALDOA	CRC	[139]
Apigenin	GLUT1, HK2, PKM2, HIF-1*α,* and PI3K-Akt-mTOR	PAC, CC, OSCs, HCC, and MEL	[140–142]
Shikonin	PKM2	LC and MEL	[143]
*Carpesium abrotanoides* (L.) root	PKM2, MCT4, and GLUT1	BRAC and MEL	[144]
Nobiletin	PGK	OSCC	[14]
Epigallocatechin	LDH	Cancer cell	[145]
Wogonin	GLUT1, HK2, PDHK1, LDHA, HIF-1*α*, and PI3K-Akt	CRC, OC, and HCC	[146, 147]
Scutellarin	PKM2	CC and CRC	[148, 149]
Matrine	HK2 and HIF-1*α*	ML and CRC	[150, 151]
Costunolide	HK2	Hepatic stellate cells	[152]
Spatholobus suberectus	LDHA	BC	[153]
Oleanolic acid	PKM2 and HIF-1*α*	Glioma and GC	[154, 155]
Silybin	GLUT4	CRC	[156]
Prosapogenin A	STAT3, GLUT1, HK, and PFK	CC, HCC, and BC	[157]
Resveratrol	GLUT1, HIF-1*α*, PFK-1, and Akt	LC, CRC, OC, NSCLC, and BC	[158–161]
Deguelin	HK2 and Akt	NSCLC	[162]
Ginsenoside 20 (S)-Rg3	STAT3 pathway	OC	[163]
Betulinic acid	LDHA and PDK1	BC	[164]
Tanshinone IIA	GLUT1, PKM2, and HK2	CC	[165]
Realgar	HIF-1*α* and PI3K-Akt-mTOR	LC	[166]
Huaier	PI3K-AKT-HIF-1*α*	LC	[167]
Cardamonin	HIF-1*α*	BC	[168]
Coptisine	circCCT3	HCC	[169]
Ginsenoside compound K	HIF-1*α*	LC	[170]
Atractylenolide I	AKT-mTOR	CRC	[171]
Licochalcone A	Akt-HK2 and PI3K-Akt-mTOR	GC and BRAC	[172, 173]

## Data Availability

No data were used to support this study.

## Abbreviations

TCM: Traditional Chinese medicine
TME: Tumor microenvironment
ROS: Reactive oxygen species
NADPH: Nicotinamide adenine dinucleotide phosphate
TCA: Tricarboxylic acid
HIF-1*α*: Hypoxia-inducible factor 1-alpha
PI3K: Phosphatidylinositol 3-kinase
mTOR: Mechanistic target of rapamycin
GLUT: Glucose transporter
SGLT: Sodium-dependent glucose transporters
HK: Hexokinase
G-6-P: Glucose-6-phosphate
GPI: Glucose-6-phosphate isomerase
F6P: Fructose-6-phosphate
PFK: Phosphofructokinase
PFKP: PFK-platelet
PFKM: PFK-muscle
PFKL: PFK-liver
F-2,6-BP: Fructose-2,6-bisphosphatase
F-2,6-BP: Fructose-1,6-bisphosphate
ALDO: Aldolase
TPI: Triose-phosphate isomerase
DHAP: Dihydroxyacetone phosphate
G3-P: Glyceraldehyde-3-phosphate
GAPDH: Glyceraldehyde-3-phosphate dehydrogenase 1,3-BPG: 1,3-biphosphoglycerate
3-PG: 3-Phosphoglycerate
ERK: Extracellular regulated protein kinases
PGK: Phosphoglycerate kinase
PGAM1: Phosphoglycerate mutase1
ENO1: Enolase1
PEP: Phosphoenolpyruvate
PKM1: Pyruvate kinase isoform1
LDHA: Lactate dehydrogenase A
LDHB: Lactate dehydrogenase B
MCT: Monocarboxylate transporter
TCA: Tricarboxylic acid
CRC: Colorectal cancer/colon cancer
HCC: Hepatocellular carcinoma
BC: Breast cancer
BRAC: Breast adenocarcinoma
TNBC: Triple-negative breast cancer
GBM: Glioblastoma
GC: Gastric cancer
LC: Lung cancer
RC: Rectal cancer
PCA: Pancreatic cancer
MEL: Melanoma
NSCLC: Non-small-cell lung cancer
OSCC: Oral squamous cell carcinoma
OSCs: Osteosarcoma cells
CC: Cervical cancer
CAC: Cervical adenocarcinoma
PAC: Prostate adenocarcinoma
BCA: Bladder cancer
GC: Gastric cancer
ACC: Adenoid cystic carcinoma
OC: Ovarian cancer
ML: Myeloid leukemia
OS: Overall survival
EGFR: Epidermal growth factor receptor
EMT: Epithelial-mesenchymal transition
CCND1: Recombinant cyclin D1
NAD+: Nicotinamide adenine dinucleotide
CAFs: Cancer-associated fibroblasts
TAMs: Tumor-associated macrophages
DCs: Dendritic cells
M-CSF: Macrophage colony-stimulating factor
GM-CSF: Granulocyte-macrophage colony-stimulating factor
G-CSF: Granulocyte colony-stimulating factor
MDSCs: Myeloid-derived suppressor cells
NK: Natural killer
PD-1: Programmed cell death protein 1
PPP: Pentose phosphate pathway
MiRNAs: Micro-RNAs
LncRNAs: Long noncoding RNAs.
